# Plant biotechnology: use of tissue culture techniques in species *Boerhavia paniculata Rich *and *Crinum americanum L *as alternative for the production of new drugs *in vitro*

**DOI:** 10.1186/1753-6561-8-S4-P243

**Published:** 2014-10-01

**Authors:** Silvana Araújo, Alberdan Santos

**Affiliations:** 1 Universidade Federal do Pará, Belém, PA, Brazil

## Background

*Crinum americanum L*. popularly known as lily is an ornamental plant that belongs to the family Amaryllidaceae alkaloids presenting with anti-inflammatory, antimicrobial and inhibition acetilcolinisterase. The species *Boerhavia paniculata Rich *is a plant from the family Nyctaginaceae popularly known as "Pega Pinto" that produces isoflavones that have action antiinflmatórias urinary tract and with its concentration in the roots. Aimed at the production and accumulation of these substances in vitro, this work aimed to study the induction of roots of the species *Boerhavia paniculata Rich *and callus induction of the species *Crinum americanum L *through the techniques of tissue culture protocols developed cell culture for production of these metabolites and evaluate the qualitative HPTLC by these cells [1,2,5]

## Methods

For callus induction species *Crinum americanum L *bulbs explants were cultured on MS medium supplemented with 3% sucrose, 0.8% agar, pH adjusted to 5.8 and 2.26 µM of 2,4-D. The flasks were kept in a chamber (BOD Eletrolab) for 30 days in the dark at 24°C, where after this period subcultures were performed in mesams conditions, totaling three subcultures. *Boerhavia paniculata Rich *seed species were germinated on rich medium containing 1.5% glucose, 0.8% agar in the counter 28°C. The roots of seedlings grown *in vitro *were inoculated in liquid MS medium containing 3% glucose, 2.46 µM of IBA and pH 5.8. The vials were kept in partial light at a temperature of 28ºC ± 2ºC. For qualitative analysis of the chemical profile of the samples was evaluated *in vitro *comparisons by HPTLC with fresh samples to assess the production potential of the metabolites of interest through the visualization of bands in UV irradiation chamber at wavelengths 365-254nm and after development reagent specific classes of metabolites: alkaloids and Dragendorff to vanillin sulfuric solution for isoflavone. Each staining bands were obtained possibility of the presence of these substances. They applied approximately 5 μL of hexane extracts samples from the chromatoplates *Boerhavia paniculata Rich *and eluted in 20 mL of Acetate / Hexane (9:1). For samples *Crinum americanum L *5 μL was used and eluted with methanolic extract in a system chloroform/ methanol (4:1), all tests were conducted at a temperature of 25°C [2-4].

## Results and conclusion

The results showed that roots of *Boerhavia paniculata Rich *obtained in vitro showed satisfactory results with IBA 2.46 µM for induction of roots. For the species *Crinum americanum L*. callus induction MS medium containing 2.26 µM of 2,4-D showed induction potential in these culture conditions. From a qualitative HPTLC chromatographic profile of the samples *in vitroBoerhavia paniculata Rich *and *Crinum americanum L*. was possible to assert that the objective was achieved, where the plates containing the spots observed at 365-254nm and after development, suggest the presence of possible chemical constituents of the class of alkaloids and isoflavonoids.
